# Diagnosis and Management of Multifocal Motor Neuropathy in the United Kingdom: A Multicentre Survey

**DOI:** 10.1111/jns.70018

**Published:** 2025-04-10

**Authors:** Yusuf A. Rajabally, Christina Englezou, Grace Cluett, Roberto Bellanti, Simon Rinaldi, Mow Wei Chin, Robert Hadden, Madalina Roman, Channa Hewamadduma, Ashleigh Murray, Amar Elsaddig, Tim Lavin, Oliver Cousins, Niranjanan Nirmalananthan, Joumana Freiha, Chinar Osman, Matthew Evans, Aisling Carr, James K. L. Holt

**Affiliations:** ^1^ Aston Medical School, Aston University Birmingham UK; ^2^ Inflammatory Neuropathy Clinic, Department of Neurology University Hospitals Birmingham Birmingham UK; ^3^ Department of Neurology The Walton Centre Liverpool UK; ^4^ Department of Neurology Oxford University Hospitals Oxford UK; ^5^ Department of Neurology King's College Hospital London UK; ^6^ Sheffield Institute of Translational Neurosciences, University of Sheffield Sheffield UK; ^7^ Manchester Centre for Clinical Neurosciences, Salford Royal Hospital Salford UK; ^8^ Atkinson Morley Neurosciences Centre St. George's University Hospitals London UK; ^9^ Wessex Neurological Centre, University Hospital Southampton Southampton UK; ^10^ MRC Centre for Neuromuscular Diseases, National Hospital of Neurology and Neurosurgery London UK

**Keywords:** conduction block, electrophysiology, intravenous immunoglobulin, multifocal motor neuropathy, outcome measures, subcutaneous immunoglobulin

## Abstract

**Background:**

We conducted a survey to determine the current diagnosis and treatment of multifocal motor neuropathy (MMN) in the United Kingdom.

**Methods:**

Demographic, diagnostic and treatment data were collected at nine UK neuroscience centres.

**Results:**

Ninety‐five subjects were included. Mean age at diagnosis was 49.9 years (SD: 11.4). Males were more commonly affected (ratio: 1.9:1). Diagnostic delay was > 1 year from the time of first neurological assessment, in > 50% of subjects. Applying modified EFNS/PNS 2010 criteria, 69/95 (72.6%) had definite MMN, 10/95 (10.5%) had probable MMN, 15/95 (15.8%) had possible MMN, through treatment responsiveness in 9/15 (60%) and 1/95 (1.1%) did not meet criteria. Cerebrospinal fluid examination, anti‐GM1 antibody testing and brachial plexus magnetic resonance imaging were non‐contributory. Immunoglobulin response was reported in 90/92 subjects (97.8%), and 84/90 (93.3%) remained on treatment after a mean of 9.4 years, at a mean dose of 26.2 g/week (range: 4–114). Mean long‐term immunoglobulin dose was 30%–60% higher than reported in neighbouring countries. Contrasting with previous reports of frequent loss of immunoglobulin response and functional decline, our physician‐assessed long‐term outcome was favourable (stable or improving) in 74/84 (88.1%) treated subjects.

**Interpretation:**

MMN diagnosis and treatment in the United Kingdom are comparable to that of neighbouring countries and follow existing guidelines. Diagnostic delay after the first neurological assessment is considerable. Electrophysiology shows at least one definite/probable conduction block in nearly 90% of cases. The mean long‐term immunoglobulin dose is higher in the United Kingdom than reported elsewhere, although highly variable. Whether higher doses of immunoglobulin may improve long‐term outcomes requires further study.

## 1 Introduction

Multifocal motor neuropathy (MMN) is a rare pure motor immune‐mediated neuropathy, producing muscle weakness and functional disability. MMN is characterised by multifocal motor deficits affecting predominantly the distal upper limbs, accompanied by hypo/areflexia, mild/no muscle wasting in the early stages, with accompanying cramps and fasciculations [1, 2]. Electrophysiologically, persistent motor conduction blocks (CBs) are detected, and IgM anti‐ganglioside antibodies to GM1 are reported in 30%–50% of affected subjects [1]. Neuroimaging through magnetic resonance (MR) neurography and ultrasonography (US) may be diagnostically helpful in revealing nerve enlargement and/or contrast enhancement [3, 4]. MMN is responsive to immunoglobulins, which remain its only licensed treatment to date [5, 6].

Few diagnostic criteria have been published for MMN. The European Federation of Neurological Societies/Peripheral Nerve Society (EFNS/PNS) Guidelines were initially published in 2006 [7] and updated in 2010 [8]. The 2010 version included a supplementary diagnostic sub‐category, ‘possible’ MMN, in addition to the ‘definite’ and ‘probable’ MMN sub‐categories. ‘Possible’ MMN allowed for the absence of detectable CB if an immunoglobulin response was present. Supportive criteria in the latest version included an elevated cerebrospinal fluid (CSF) protein level of < 1 g/L, in addition to three of the 2006 version, that is, presence of anti‐GM1 antibodies, MR imaging (MRI) abnormalities of the brachial plexus, and improvement with intravenous immunoglobulins (IVIg). Furthermore, two supportive criteria were required instead of one to diagnose ‘probable’ MMN if only one nerve met electrophysiological criteria. On the other hand, elevated CSF protein > 1 g/L was removed from the exclusion criteria in the 2010 version.

The uptake and consequent value of EFNS/PNS Guidelines for inflammatory neuropathies at large, and for MMN in particular, were found to be disappointingly low in clinical practice in an international audit [9]. Improving clinical relevance and interest hence represents a challenge for the future European Academy of Neurology/Peripheral Nerve Society (EAN/PNS) Guidelines update for MMN, planned for 2025–2026. In England, the Commissioning Policy for the use of immunoglobulin, published by the National Health Service England (NHSE), requires for treatment approval, a diagnosis of MMN made by a neurologist, ‘with or without CB’ and causing ‘significant functional impairment inhibiting normal daily activities’ [10]. Fulfilment of diagnostic criteria is not required. Although such flexibility may be helpful in routine practice, the lack of application of strict diagnostic criteria poses problems, particularly in MMN, where (i) several disease mimics exist, (ii) multiple factors may impact the use of outcome measures to evaluate treatment effects, leading to possible erroneous diagnostic confirmation and inappropriate continuation of a high‐cost therapy not devoid of possible serious side‐effects. The NHSE Commissioning Policy recommends dose reductions and increases in treatment interval to the minimum effective as well as periodic trials of treatment cessation, although without specific advice for MMN [10].

The British Peripheral Nerve Society (BPNS) proposed to conduct a survey of a sample of its members running peripheral nerve clinics to ascertain the current state of diagnostic and therapeutic practice for subjects affected by MMN in the United Kingdom. We aimed (i) to determine in what proportions a modified form of EFNS/PNS 2010 diagnostic criteria applied to our cohort were fulfilled, particularly with regard to electrodiagnosis and other available investigative methods, (ii) to ascertain the treatment modalities as well as monitoring methods utilized, (iii) to compare UK practice with that reported in similar cohorts in neighbouring countries and with existing disease guidelines, and (iv) to gain insight into areas where improvement may be desirable and achievable for diagnosis, monitoring and treatment, and where further research may be helpful.

## 2 Materials and Methods

A web‐based survey was conducted through an e‐mail invitation by the BPNS to consultant neurologists running peripheral nerve services in 17 selected UK neuroscience centres. The survey was open for 12 months, between October 2023 and September 2024. Anonymised data were requested from treating clinicians for each included patient with a clinical diagnosis of MMN.

The treating clinician or their team made the initial diagnosis and the decision to include. Inclusion required fulfilment of clinical criteria for MMN, defined as per EFNS/PNS Guidelines of 2010, as ‘slowly progressive or stepwise progressive, focal asymmetric limb weakness, with motor involvement in the nerve distribution of at least two nerves for more than a month’, with the mandatory absence of all four exclusion clinical criteria (sensory features, upper motor neuron signs, bulbar weakness, diffuse symmetric weakness at presentation). Detailed onset and current clinical descriptions were not requested from participating clinicians. Clinicians were asked to provide demographic details, age at diagnosis and diagnostic delay in relation to the time of first neurological assessment. Electrophysiological results were evaluated as per a modified version EFNS/PNS 2010 electrodiagnostic criteria, pragmatically based (i) on percentage differences in compound muscle action potential (CMAP) amplitude rather than areas as more readily available from reports (ii) inclusion of motor conduction studies of radial, musculocutaneous and axillary nerves, in addition to those of median, ulnar and common peroneal nerves. CMAP amplitude percentage reductions of > 50% defined definite CB, and > 30% defined probable CB. In addition, details were requested on number, site and degree of CBs, number of electrodiagnostic studies performed, CSF findings, anti‐GM1 antibody positivity, results of MR of the brachial plexus and nerve US, outcome measures used for disease monitoring, immunoglobulin treatment response ascertained through improvement on any utilized outcome measure of any amplitude deemed relevant by the treating clinician, immunoglobulin administration method and maintenance dosage, and use of alternative treatments. The modified EFNS/PNS 2010 criteria diagnostic sub‐category was ascertained in each case. Physician‐assessed long‐term outcome (‘stable after initial improvement’, ‘sustained improvement’ or ‘deterioration after initial improvement’) was established for each subject on continuing therapy. Cases lacking demographic data, electrophysiological data confirmatory of diagnostic sub‐category, use of at least one outcome measure, or clear description of response or lack of response to treatment, were excluded.

Each contributing centre obtained local governance approval to perform a clinical practice evaluation and, as such, UK ethics committee approval was not required. All patient‐identifiable data collected at participating centres were removed prior to submission of survey responses to the BPNS. Hence, all data received and analysed by the BPNS survey organisers (J.K.L.H., C.E. and Y.A.R.) were fully anonymised.

Statistical analyses were performed with SPSS 28.0 (Armonk, USA). A comparison of proportions was performed using Fisher exact tests, and a comparison of means was performed using independent *t*‐tests. Correlations were performed by Spearman's rank correlation tests. Significance was set at *p* < 0.05 for all tests.

## 3 Results

Nine of the 17 centres (52.9%) responded to the survey invitation. Data were submitted for 167 subjects. Due to insufficient provision of the mandatory demographic, electrophysiological, or treatment details, 72 subjects were excluded from the analysis.

The main characteristics of the 95 included subjects are summarised in Table 1. There were 62 males and 33 females (ratio 1.9:1). The mean age at diagnosis was 49.9 years (SD: 11.4), and the mean age at the time of the survey was 59.3 years (SD: 12.6). The mean current age of males was lower than that of females (57.2 years vs. 63.3 years; *p* = 0.024). The mean disease duration during the follow‐up period was 9.4 years (SD: 6.8). Six of the 95 subjects (6.3%) had concurrent diabetes, and 6 of the 95 (6.3%) had another associated autoimmune disorder. Time to diagnosis from the first neurological assessment was < 3 months in 21/95 subjects (22.1%), < 6 months in 32/95 (33.7%) and < 12 months in 44/95 (46.3%). Diagnostic delay from the first neurological assessment was ≥ 1 year in 51/95 subjects (53.7%), > 3 years in 21/95 (22.1%) and > 5 years in 10/95 (10.5%).

**TABLE 1:** Characteristics of 95 subjects with a clinical diagnosis of MMN from 9 UK centres.

Mean current age, years (SD)	59.3 (12.6)
Mean age at time of diagnosis, years	49.9 (11.4)
Gender F:M (ratio)	33:62 (1:1.9)
Mean follow‐up duration, years (SD)	9.4 (6.8)
Rate of associated diabetes (%)	6/95 (6.3%)
Rate of other associated autoimmune disorders (%)	6/95 (6.3%)

Main motor electrophysiological findings are summarised in Table 2. Nerve conduction studies had been repeated once in 48/95 subjects (50.5%), and more than once in 18/95 (18.9%). Definite or probable CB was reported for at least one motor nerve in 85/95 subjects (89.5%), and for at least two motor nerves in 50/95 (52.6%). At least one definite CB was detected in 70/95 subjects (73.7%), and at least one probable CB in 15/95 (15.8%). The mean number of definite or probable CBs identified per patient was 1.70 (SD: 1.11; range: 0–5). CBs were detected most commonly on the ulnar nerve (70/165; 42.4%), followed by the median nerve (58/165; 35.2%) and the radial nerve (21/165; 12.7%). CBs were rarely reported on the common peroneal nerve (6/165; 3.6%), musculocutaneous nerve (2/165; 1.2%) and axillary nerve (2/165; 1.2%).

**TABLE 2:** Electrophysiology of 95 subjects with MMN from 9 UK centres, considering definite conduction block (CB) as > 50% CMAP amplitude reduction and probable CB as > 30% CMAP amplitude reduction.

Number of subjects with at least one definite or probable CB (%)	85/95 (89.5%)
Number of subjects with at least two definite or probable CBs (%)	50/95 (52.6%)
Number of subjects with at least one definite CB (%)	70/95 (73.7%)
Number of subjects with at least one probable CB (%)	15/95 (15.8%)
Mean number of CBs per patient (SD)	1.70 (1.11)

Twenty‐four of the 95 subjects (25.3%) had a CSF study. CSF protein was elevated above the range for the local laboratory but < 1 g/L in 6/24 (25%), and normal in 17/24 (70.8%). In one subject, CSF protein was 1.5 g/L. Anti‐ganglioside antibody testing was performed in 75/95 subjects (78.9%), and IgM anti‐GM1 antibodies were detected in 17/75 (22.7%). MRI of the brachial plexus was performed in 24/95 subjects (25.3%) and reported as abnormal in 4/24 (16.7%). Nerve US was performed in 2/95 subjects (2.1%), with nerve enlargement reported in both cases.

The fulfilment of modified EFNS/PNS 2010 criteria by the 95 studied subjects is summarised in Table 3. Sixty‐nine of 95 subjects (72.6%) had definite MMN, 10/95 (10.5%) had probable MMN, 15/95 (15.8%) had possible MMN, and 1/95 (1.1%) did not meet the criteria. CSF examination, anti‐GM1 antibody testing, and brachial plexus MRI did not contribute to the diagnosis in any of the 94 subjects meeting the criteria. A documented immunoglobulin response enabled, on the other hand, 9/10 subjects (90%) without detectable CB to be classified as possible MMN.

**TABLE 3:** Fulfilment of modified EFNS/PNS 2010 criteria by 95 subjects from 9 UK centres with a clinical diagnosis of MMN.

	Modified EFNS/PNS criteria for MMN (2010)
Definite MMN (%)	69/95 (72.6%)
Probable MMN (%)	10/95 (10.5%)
Possible MMN (%)	15/95 (15.8%)
Not MMN (%)	1/95 (1.1%)

Ninety‐two subjects were treated with a trial of IVIg. Three were left untreated as considered stable and not significantly disabled. Ninety of the 92 treated subjects (97.8%) were fully or partially immunoglobulin‐responsive. Eighty‐four of the 90 immunoglobulin responders (93.3%) were on continuing treatment at the time of the survey, with IVIg in 68/84 (81%) and subcutaneous immunoglobulin (SCIg) in 16/84 (19%). Latest mean frequency of administration was 4.2 weeks (SD: 3.2; median: 4) but varied widely, from twice weekly to once every 20 weeks. Latest mean weekly immunoglobulin dose was 26.2 g (SD: 17.1), with a wide range (4–114 g). Mean weekly immunoglobulin dosage varied widely in‐between centres, ranging from 8.2 to 50 g. Physician‐evaluated long‐term outcomes were ‘stable after initial improvement’ in 43/84 (51.2%), ‘sustained improvement’ in 31/84 (36.9%) and ‘deteriorating after initial improvement’ in 10/84 (11.9%). There were no correlations between the latest immunoglobulin dose with physician‐evaluated long‐term outcome (*p* = 0.44), age at diagnosis (*p* = 0.93), diagnostic delay from first neurological assessment (*p* = 0.23) or modified EFNS/PNS 2010 diagnostic sub‐category (*p* = 0.89).

Immunosuppressants were used in 3/95 subjects (3.2%), with mycophenolate mofetil in one, mycophenolate mofetil and azathioprine in one, and natalizumab in one. All three subjects had been immunoglobulin responsive and were on continuing treatment. A beneficial effect was only described in the subject on mycophenolate mofetil alone, with a subsequent reduction of the immunoglobulin dose.

The most common outcome measure used to evaluate treatment effects was the MMN‐Rasch‐built Overall Disability Scale (MMN‐RODS), in 74/95 subjects (77.9%), followed by the MRC sum score in 73/95 (76.8%), and grip strength by Jamar dynamometer or Martin vigorimeter in 51/95 (53.7%). Other outcome measures used were the Overall Neuropathy Limitation Scale (ONLS) in 46/95 (48.4%), the timed 10‐m walk test in 30/95 subjects (31.6%), the 9‐hole Peg Test in 18/95 (18.9%), the Inflammatory Rasch‐built Overall Disability Scale (I‐RODS) in 11/95 (11.6%), pinch strength in 6/95 (6.3%) and the Inflammatory Neuropathy Cause and Treatment (INCAT) scale in 3/95 (3.2%).

## 4 Discussion

This survey from nine UK centres demonstrates many similarities with previous reports from other patient cohorts. We found a comparable gender ratio and a lower mean age in male subjects, as previously reported by Cats et al. in the Dutch MMN population [11]. Although not strictly comparable due to differences in methodology, the diagnosis of MMN in the United Kingdom remains delayed in 2023–2024, with > 50% of diagnoses made > 12 months after the first neurological assessment, which may suggest a lack of familiarity of non‐specialist neurologists with the clinical presentation of MMN. In the Netherlands in 2001–2006, the median time to diagnosis was 2 years from symptom onset, and in Austria in 2018 [12], the median time was 3 years. Unfortunately, the time to diagnosis from symptom onset was not available in our cohort.

Although we considered CMAP amplitudes rather than areas, our electrophysiological findings were comparable to those of the Dutch study, for the percentage of patients with at least one definite CB (72.6% vs. 81%) and with at least one probable CB (15.8% vs. 21%) [11]. The Austrian study also reported similar results (82.5% with at least one definite CB and 12.3% with at least one probable CB), producing comparable rates of the fulfilment of EFNS/PNS 2010 diagnostic sub‐categories as those observed in our survey [12]. In contrast, Doneddu et al. reported that only 26%, 35% and 26% of their Italian cohort met the requirements for definite, probable, and possible MMN, respectively [13]. The reasons for these differences are uncertain but may in part relate to the degree of extensiveness of electrophysiological testing. Of note, in the Italian cohort, use of CMAP amplitude instead of area, through the application of American Association of Electrodiagnostic Medicine (AAEM) criteria [14] improved sensitivity for definite and probable sub‐categories. The differences between CB evaluation through amplitude vs. area evaluation remain uncertain. Direct prospective comparative studies in diseased subjects of sensitivity and specificity vs. healthy controls have not, to our knowledge, been performed. The EFNS/PNS Guidelines opted for area over amplitude based on animal studies and computer modelling, which indicated that reductions of up to 50% of the CMAP on proximal stimulation might be exclusively due to interphase cancellation [15], although acknowledged the lack of evidence [8]. Similarly, studies on human nerves have shown the dependency of CB detection on the degree of distal and proximal CMAP dispersion [16]. Novel methods using deconvolution resulting in optimally reconstructed distal and proximal CMAPs have been proposed to include consideration of temporal dispersion in CB evaluation but have not been applied in clinical practice and remain of unclear practical value [17, 18]. Hence, there remains, in summary, little evidence to question amplitude comparisons in CB evaluation, which we used given its wide and predominant use in clinical practice in the United Kingdom. In addition, we found, despite this difference in methods, that CB was detected in similar proportions to the Austrian study for ulnar nerves (42.4% vs. 35.1%), median nerves (35.2% vs. 33.3%) and radial nerves (12.7% vs. 12.6%). Of note, electrophysiology was repeated in the majority of our subjects, with the most informative study included.

The rate of IgM anti‐GM1 antibody positivity in our series was lower than reported in the Netherlands (43%) [19], Austria (43%) [12] and Japan (54%) [20]. However, our rate was comparable to that of a previous UK study (25.5%) [21] and that of an earlier Italian study (29.2%) [22]. As opposed to other recent studies [13], anti‐GM1 antibody positivity had no impact upon diagnosis in our cohort. MRI of the brachial plexus, performed in one out of four patients and reported as abnormal in only one out of six patients, was similarly globally unhelpful. This may be due to access to brachial plexus MRI in the United Kingdom, as well as to the previously reported low reliability of qualitative MRI evaluation [23]. Nerve US is rarely performed in the United Kingdom, despite its potential in MMN and reported superiority vs. MRI [24]. CSF protein levels were, finally, non‐contributory, as also reported by the recent Italian study [13]. We believe the lack of contribution of supportive criteria besides treatment response to diagnosis in our cohort relates mainly to the high CB detection rate and sensitivity of electrophysiology rather than the low sensitivity of these tests, as only a limited number of subjects had to undergo these additional investigations.

The mean current weekly immunoglobulin dose was 26.2 g in our series, with wide variations in‐between patients and in‐between centres. This is higher than those previously reported in cohorts from Italy (19.2 g) [25], the Netherlands (17 g) [11], Austria (about 16 g) [12] and Denmark (20.0 g) [26]. Of note, over a follow‐up period of more than 9 years, physician‐assessed deterioration after initial improvement occurred in less than 12% of subjects in our cohort. This contrasts with the gradual decline of strength and function back to pre‐treatment levels after initial amelioration, commonly reported previously in other series. In the Austrian study, the physician‐reported clinical global impression showed that a majority of patients no longer responded to treatment and > 20% deteriorated despite treatment, after a median of 7.47 years of follow‐up [12]. A large Dutch study of clinical outcomes similarly demonstrated decline despite treatment on 7/10 objective measures [27], as did a small Malaysian study after dose IVIg reduction, with no improvement despite dose re‐increase [28]. On the other hand, it is noteworthy that our results resemble those of an earlier US study, which demonstrated preserved muscle strength and function over 7.5 years, with continuing high doses of immunoglobulin, of a mean of 1.63 g per kilogram every 4 weeks, corresponding to 30.6 g weekly, assuming a mean weight of 75 kg [29].

Despite its multiple advantages [30], SCIg remains rarely used in the United Kingdom (19%), as is also the case in Austria (11%) [12]. This may be due to insufficient specialist nursing support, resulting in poor access to training for patients to self‐administer SCIg. Use of immunosuppressant treatment was exceptional in our cohort, in line with guideline recommendations [8]. It is possible the favourable outcomes described by the surveyed physicians may represent part of the explanation for this, as a Good Practice Point of the guidelines only mentions immunosuppressant use if IVIg is not sufficiently effective, despite the absence of evidence. Six of the 90 immunoglobulin‐responsive subjects of our cohort (6.7%) had discontinued treatment. This is in keeping with the high immunoglobulin dependency rates reported in MMN in different cohorts [19, 20, 31]. Unfortunately, clinical details regarding progression in the minority of our patients who stopped treatment were not available.

Although multiple outcome measures were used by surveyed clinicians, the MMN‐RODS, a disease‐specific disability scale [32], was the most common. This scale has rarely been used in previous studies of MMN cohorts. It represents, however, arguably, the most clinically relevant scale currently available for MMN, and may give more confidence in the treatment response rates as well as physician‐evaluated long‐term outcomes of our survey. We did not determine the application of minimal clinically important difference (MCID), because this is not required for immunoglobulin prescribing by regulatory authorities in the United Kingdom and is not formally implemented in routine clinical practice.

Our survey has several limitations. Its retrospective design, based on e‐mail invitations of selected major UK neuroscience units, represents a selection bias, further complicated by possible selective reporting at each centre. The survey response rate was low (9/17 centres; 52.9%), and > 40% of subjects reported had to be excluded due to incomplete data. In addition, detailed clinical phenotype and outcome measures pre‐ and post‐treatment were not available. We used modified EFNS/PNS criteria considering CMAP amplitude rather than area reduction, which may have improved sensitivity [13]. Other criteria for MMN, such as those of the AAEM, were furthermore not evaluated [14]. However, the findings offer insight into the current management of MMN in the United Kingdom through the evaluation of a large cohort, and illustrate the similarities and differences with other countries, and areas in need of improvement and/or requiring further study.

In conclusion, the diagnosis and treatment of MMN in the United Kingdom are comparable to those reported in neighbouring countries, and international guidelines are adhered to. Diagnostic delay is considerable after the first neurological assessment, and prompter clinical recognition is desirable. Nearly 90% of affected subjects had electrophysiological evidence of at least one definite or probable CB, and the added value of CSF examination, anti‐GM1 antibody testing, and nerve imaging could not be demonstrated. Although the disease‐specific MMN‐RODS scale was the most commonly used, the systematic application of MCID is an important further step required to define therapeutic response more relevantly. Finally, the mean long‐term immunoglobulin dose was 30%–60% higher in this UK cohort compared to that in neighbouring countries, with the concurrent suggestion of possible better physician‐perceived long‐term outcomes in the United Kingdom. Caution is, however, clearly required with regard to these findings and their potential implications, in the absence of available detailed pre‐ and post‐treatment outcomes. Further study of the potential role of long‐term high‐dose immunoglobulin treatment in preventing loss of treatment response and functional decline is warranted in MMN.

## Conflicts of Interest

Y.A.R. has received speaker/consultancy honoraria from Argenx, Sanofi, Grifols, Dianthus, Takeda, Johnson & Johnson, LFB, and Polyneuron, has received educational sponsorships from LFB and CSL Behring and has obtained research grants from LFB. G.C. has no disclosures. C.E. has no disclosures. R.B. has received a research grant from Grifols. S.R. has received honoraria for lectures given at the request of Excemed, Fresenius, CSL Behring, UCB, argenx, the Beijing Association of Holistic and Integrated Medicine, and the Irish Institute of Clinical Neuroscience. M.W.C. has no disclosures. R.H. has received consultancy honoraria from CSL Behring, Argenx, Dianthus and Takeda. M.R. has no disclosures. C.H. has received speaker/consultancy honoraria from Argenx, UCB, Jansen and Jansen, Roche, Biogen, Avidity, Dyne, Remgen, Novartis and Pepgen. A.M. has no disclosures. A. A. has no disclosures. T.L. has received speaker/consultancy honoraria from Alnylam and Argenx. O.C. has no disclosures. N.N. has received educational sponsorships from CSL Behring. J.F. has no disclosures. C.O. has received speaker/consultancy honoraria from Takeda and Terumo BCT. M.E. has no disclosures. A.C. has received consultancy honoraria and/or educational sponsorships from Alnylam, Argenx, Bristol Meyer Squibb, CSL Behring, Grifols, Lupin and Takeda. J.K.L.H. has received educational sponsorships from CSL Behring and consultancy honoraria from CSL Behring and Takeda.

## Data Availability

The data that support the findings of this study are available on request from the corresponding author. The data are not publicly available due to privacy or ethical restrictions.
